# Supplementary material for: Dispersed surface Ru ensembles on MgO(111) for catalytic ammonia decomposition
Source: Nat Commun. 2023 Feb 6;14:647. doi: 10.1038/s41467-023-36339-w (PMC9902439; doi:10.1038/s41467-023-36339-w)
Supplement: Supplementary file 1 — Supplementary information [file 41467_2023_36339_MOESM1_ESM.pdf]

Dispersed Surface Ru Ensembles on MgO(111) for Catalytic Ammonia Decomposition

Huihuang Fang,[1†] Simson Wu,[1†] Tugce Ayvali,[1] Jianwei Zheng,[1] Joshua Fellowes,[1] Ping-Luen Ho,[1] Kwan Chee Leung,[1] Alexander Large,[2] Georg Held,[2] Ryuichi Kato,[3] Kazu Suenaga,[3] Yves Ira A. Reyes[4], Ho Viet Thang[5], Hsin-Yi Tiffany Chen,[4,6,7] and Shik Chi Edman Tsang[1]*

*[1]The Wolfson Catalysis Centre, Department of Chemistry, University of Oxford, Oxford, OX1 3QR, UK; † These authors contributed equally; *email: edman.tsang@chem.ox.ac.uk*

[2]Diamond Light Source, Didcot, OX11 0DE, UK

[3]National Institute of Advanced Industrial Science and Technology (AIST), Central 5, 1-1-1 Higashi, Tsukuba 305-8565, Japan

[4]Department of Engineering and System Science, National Tsing Hua University, Hsinchu 300044, Taiwan

[5] The University of Danang, University of Science and Technology, DaNang 550000, Vietnam

[6]College of Semiconductor Research, National Tsing Hua University, 101, Sec. 2, Kuang-Fu Road, Hsinchu 300044, Taiwan

[7]Department of Material Science and Engineering, National Tsing Hua University, Hsinchu 300044, Taiwan

Supplementary materials

Table S1 NH_3 conversion over Ru catalysts under atmospheric pressure.

Table S2 Least square fitting for Ru/MgO(111) and Ru/MgO(110).

Table S3: Comparison of Ru/MgO catalysts regarding surface area, catalytic activity: per gram of catalyst and per unit surface area.

Table S4 Comparison of kinetic parameters of supported Ru catalysts.

Table S5 Comparison of activity for ammonia decomposition over atomic dispersed Ru/MgO(111), Ru/MgO(110) and Ru/MgO(100) with an ultra-low loading of 0.01 wt.%.

Table S6 Summary of literature experimental attributions of IR bands assigned to carbonates species adsorbed on MgO surface.

Table S7 Summary of the percentage of exposed facets over different MgO samples based on low temperature CO_2 adsorption.

Table S8 Rates of ammonia decomposition over the different MgO supported Ru catalysts.

Table S9 Rates of ammonia decomposition over the MgO(111) supported Ru catalysts with different loadings.

Fig. S1 TEM images of (a) MgO(111), (b) MgO(110) and (c) MgO(100).

Fig. S2 TEM image of Ru/MgO(111).

Fig. S3 a. A magnified STEM image of Ru/MgO(111); b. Fast-Fourier-Transform of the spectra in a, confirming the atomic arrangement of (111) facet; c. HAADF-STEM image of 3.4 wt%-Ru/MgO(111) observed from [110] direction; d. Corresponding acquisition of EELS (a) and (b) and HAADF acquired along the lines (i) and (ii); e. Atomic model was also provided for reference.

Fig. S4 HAADF-STEM images of (a) Ru/MgO(110) with clusters/nanoparticles and (b) Ru/MgO(100) with nanoparticles.

Fig. S5 HAADF-STEM images of Ru/MgO(111) observed from (a) [111], (b) [110], and (c) [100] directions; (d-f) ADF intensities acquired alone the lines in (a-c); the black or red line in (d-f) are matched with the marked black or red dash lines in (a-c).

Fig. S6 Fourier Transformed of Ru K-edge EXAFS spectra for Ru/MgO(110) and Ru(0) foil at ambient temperature. Parameters obtained from least-square fitting are shown on the right. Parameters used: k-range: 3-11.4, R-range: 1.35-3, R-factor: 2.0%.

Fig. S7 Thermodynamic equilibrium for ammonia decomposition as a function of temperature under atmosphere pressure.

Fig. S8 Dependence of ammonia decomposition rate of supported Ru samples on the partial pressures of N_2 at 350 °C.

Fig. S9 Surface reconstruction of MgO-cube into MgO-octahedron with different percentages of exposed (100), (110) and (111) facets.

Fig. S10 In situ DRIFTS of OH^- signals during the NH_3 transformation over Ru/MgO(111) under different temperatures.

Fig. S11 In situ DRIFTS study of NH_3 adsorption over Ru/MgO(100) under different temperatures.

Fig. S12 In situ DRIFTS of OH^- signals during the NH_3 transformation over Ru/MgO(100) under different temperatures.

Fig. S13 Ru 3d of AP-XPS spectra at 300 °C over Ru/MgO(100) under alternative sweeping between 0.9 mbar Ar and 0.9 mbar NH_3.

Fig. S14 O 1s of AP-XPS spectra at 300 °C over Ru/MgO(100) under alternative sweeping between 0.9 mbar Ar and 0.9 mbar NH_3.

Fig. S15a. Fourier Transformed of Ru K-edge EXAFS spectra for Ru/MgO(111) at typical 0.5% and 3% Ru after hydrogen pre-reduction, which show absence of Ru-Ru bond. Parameters obtained from least-square fitting are shown on the right. Fitting parameters for 3% Ru/MgO(111) used: k-range: 3-12, R-range: 1-3, R-factor: 1.9%.

Fig. S15b. Fourier Transformed of Ru K-edge EXAFS spectra for 3% Ru/MgO(110) and Ru(0) foil after hydrogen pre-reduction, which show presence of Ru-Ru bond. Parameters obtained from least-square fitting are shown on the right. Fitting parameters for 3% Ru/MgO(110) used: k-range: 3-11.4, R-range: 1.35-3, R-factor: 2.0%.

Fig. S15c. Fourier Transformed of Ru K-edge EXAFS spectra for 3% Ru/MgO(110) or >4% Ru/MgO(111) and Ru(0) foil after hydrogen pre-reduction in Ar, which show presence of extensive Ru-Ru bonds. Parameters obtained from least- square fitting are shown on the right. Fitting parameters for 3% Ru/MgO(110) used: k-range: 3-11.4, R-range: 1.35-3, R-factor: 2.0%.

Fig. S16a Comparison of the thermodynamic stability of a single Ru atom (A) absorbed on MgO(111) (O-O-O hollow) site and (B) embedded in place of an Mg atom of MgO(111).

Fig. S16b Comparison between DFT models of Ru atom adsorbing on stoichiometric and non-stoichiometric MgO(111).

Fig. S16c Bader charge (q, in |e|) of Ru (pink) adsorbed on MgO(111) and the change in Bader charge before and after Ru adsorption (Δq, in |e|) of MgO surface O atoms one bond (blue) and two bonds (cyan) away from Ru.

Fig. S17 Optimized structures of atomic Ru pairs adsorbed on the surface of (A) MgO(111), (B) MgO(100) and (C) MgO(110) shown from (left) side view, (right) top view. The shortest distance between two Ru single atoms on each systems are shown. Ru, Mg and O atoms are shown as purple, orange and red spheres.

Fig. S18 DFT calculated energy profile of the ammonia decomposition pathway on Ru pair sites supported on MgO(111). The N-N recombination step with a calculated activation energy value of 1.49 eV (TS5) is the rate-determining step.

Fig. S19 The catalytic performance of ammonia decomposition and stability of Ru/MgO(111) as a function of step-by-step temperature ramp down and up.

Fig. S20 Recorded DRIFTS of adsorbed species over MgO(111), MgO(110) and MgO(100) after CO_2 adsorption under Ar gas flow.

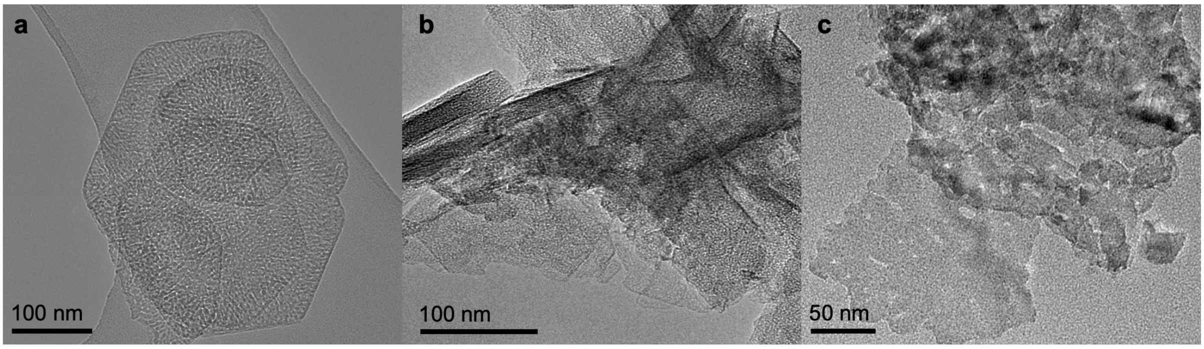

Fig. S1 TEM images of (a) MgO(111), (b) MgO(110) and (c) MgO(100).

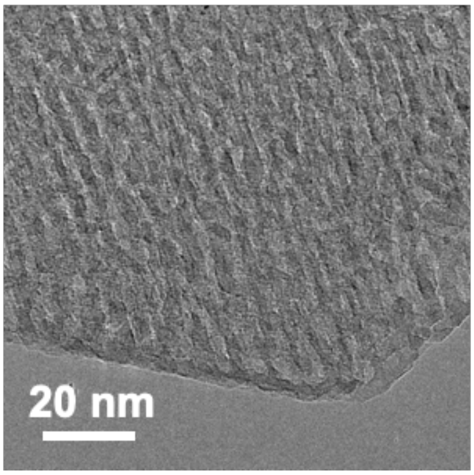

Fig. S2 TEM image of Ru/MgO(111).

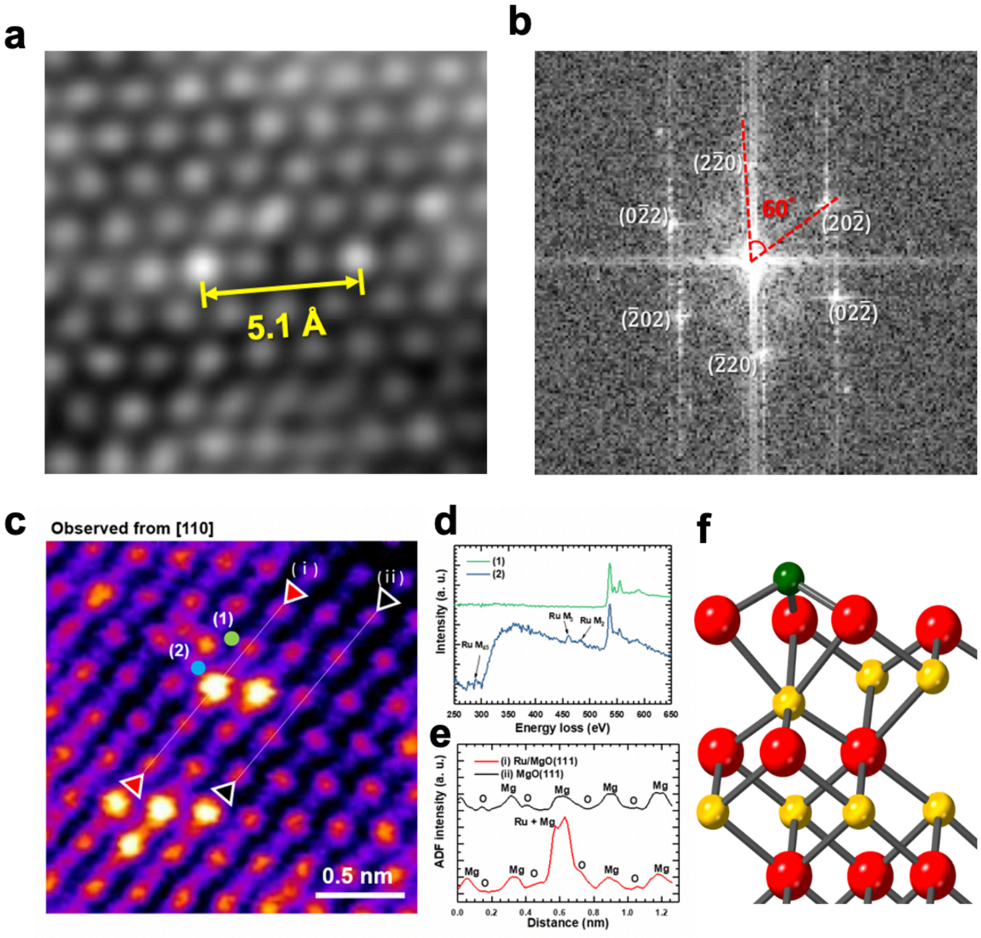

Fig. S3 A magnified STEM image of Ru/MgO(111); b. Fast-Fourier-Transform of the spectra in a, confirming the atomic arrangement of (111) facet; c. HAADF-STEM image of 3.4 wt%-Ru/MgO(111) observed from [110] direction; d. Corresponding acquisition of EELS (1) and (2) and HAADF intensity profile acquired along the lines (i) and (ii); e. Atomic model was also provided for reference.

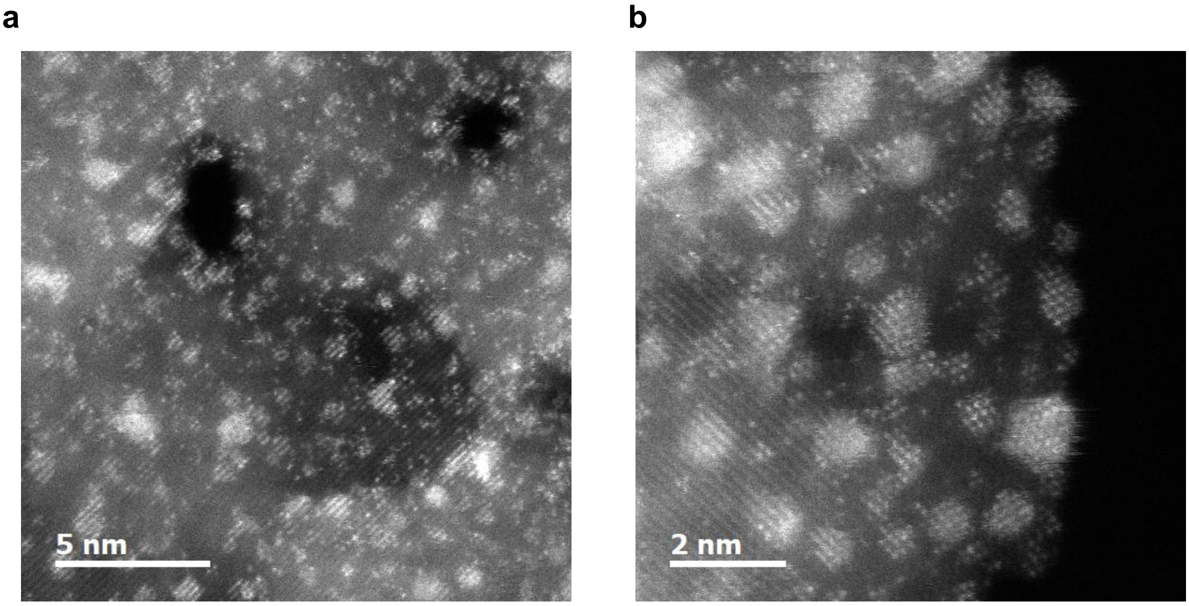

Fig. S4 HAADF-STEM images of (a) 3wt% Ru/MgO(110) with clusters/nanoparticles and (b) 3 wt% Ru/MgO(100) with nanoparticles.

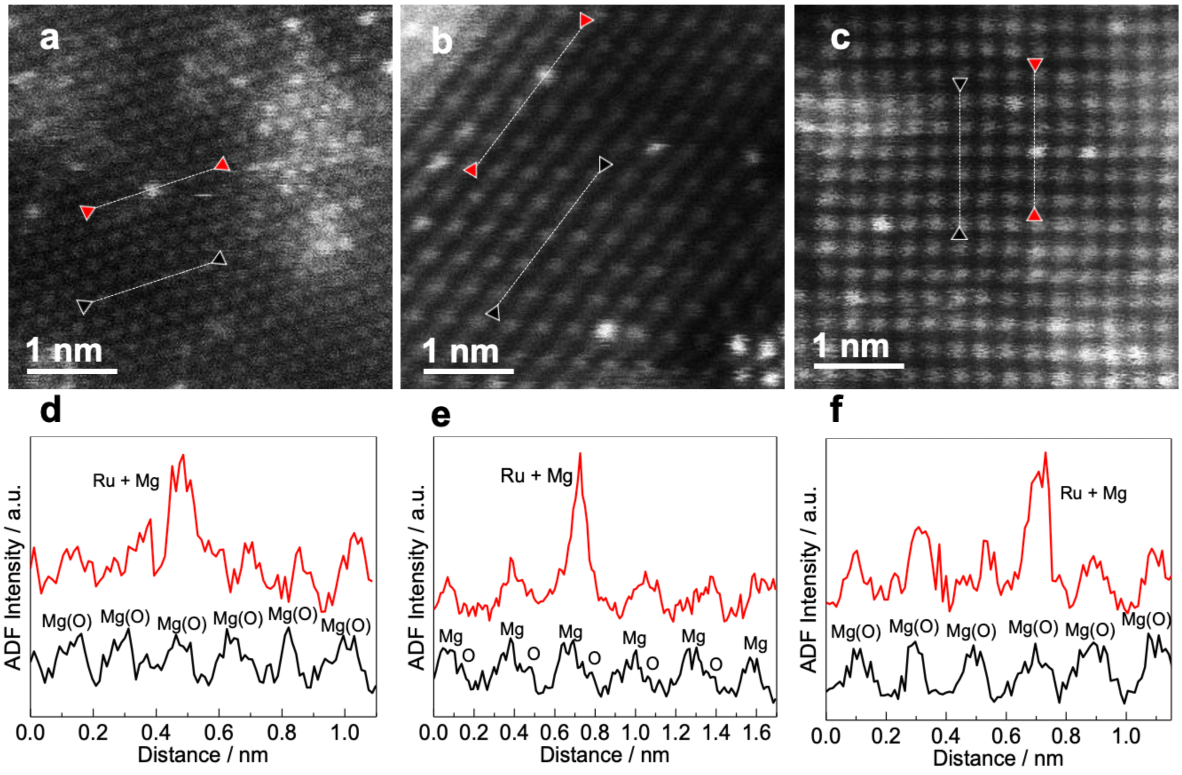

Fig. S5 HAADF-STEM images of Ru/MgO(111) observed from (a) [111], (b) [110], and (c) [100] directions; (d-f) ADF intensities acquired along the lines in (a-c); the black or red line in (d-f) are matched with the marked black or red dash lines in (a-c).

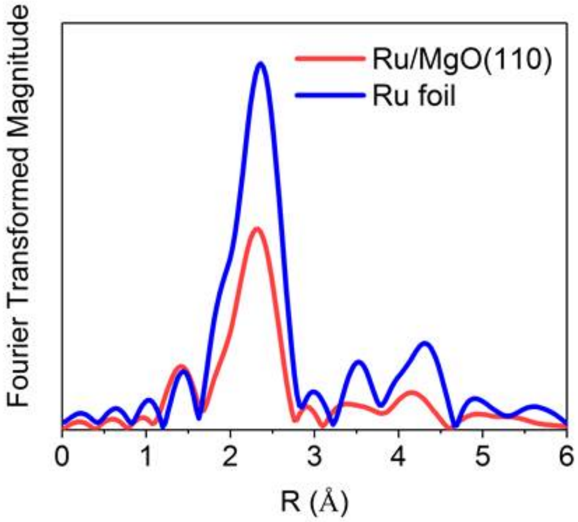

Fig. S6 Fourier Transformed of Ru K-edge EXAFS spectra for Ru/MgO(110) and Ru(0) foil in air at ambient temperature. Parameters obtained from least-square fitting are shown on the right. Parameters used: k-range: 3-11.4, R-range: 1.35-3, R-factor: 2.0%.

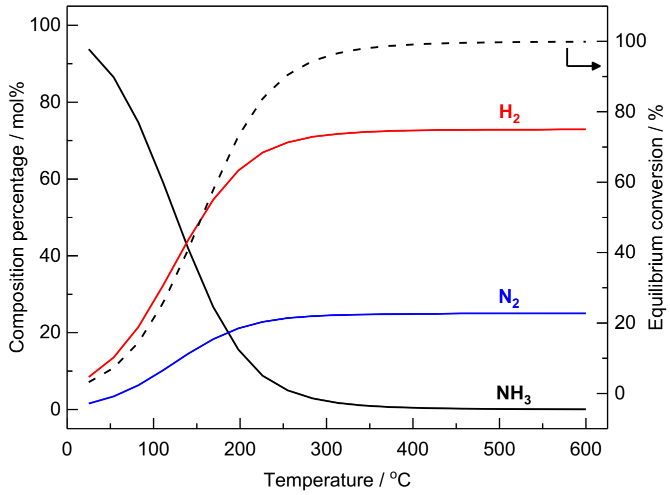

Fig. S7 Thermodynamic equilibrium for ammonia decomposition as a function of temperature under atmosphere pressure.

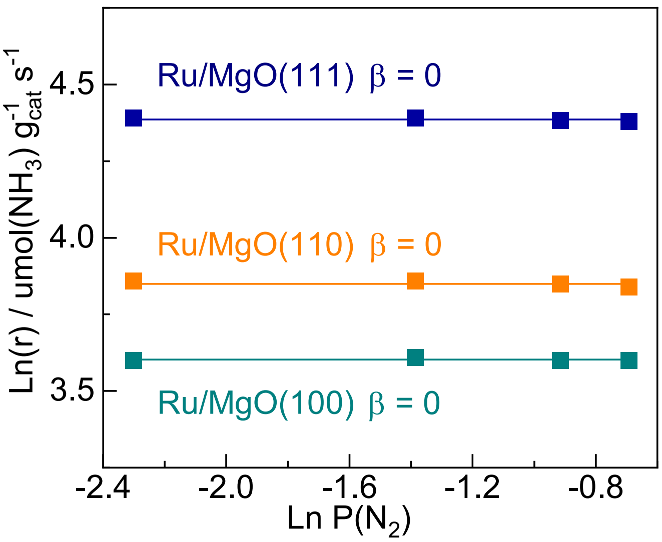

Fig. S8 Dependence of ammonia decomposition rate of supported Ru samples on the partial pressures of N_2 at 350 °C.

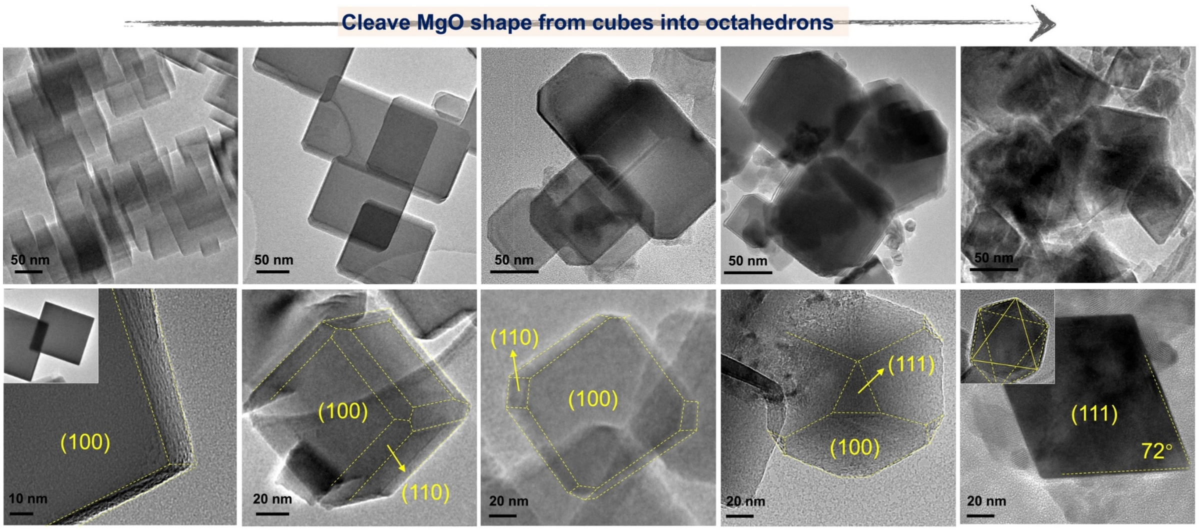

Fig. S9 Surface reconstruction of MgO-cube into MgO-octahedron with different percentages of exposed (100), (110) and (111) facets.

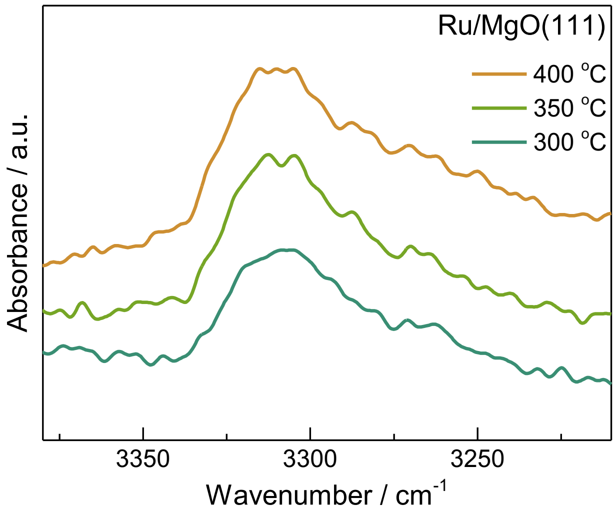

Fig. S10 In situ DRIFTS of OH^- signals during the NH_3 transformation over Ru/MgO(111) under different temperatures.

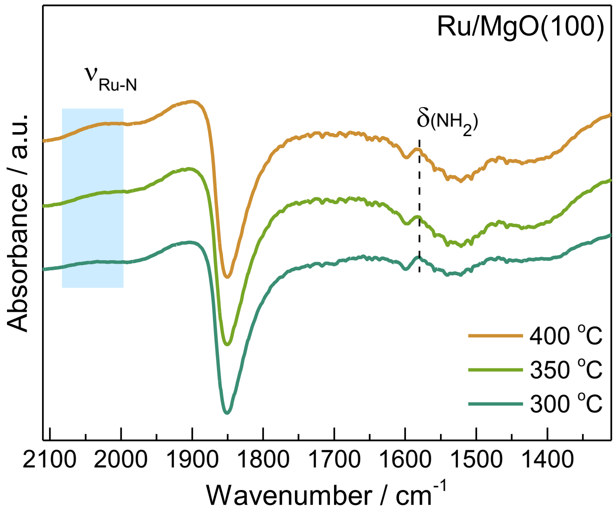

Fig. S11 In situ DRIFTS study of NH_3 adsorption over Ru/MgO(100) under different temperatures.

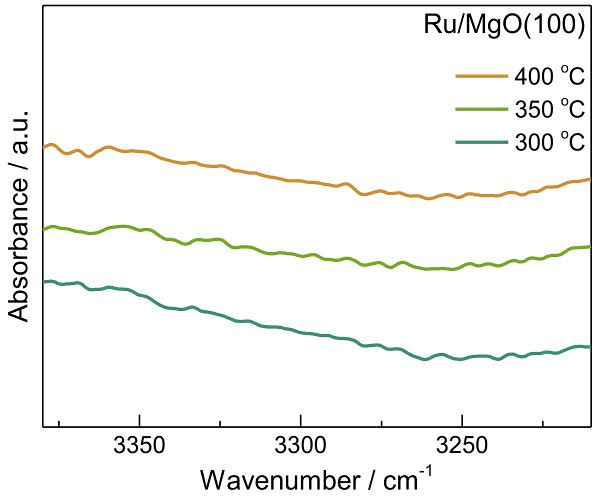

Fig. S12 In situ DRIFTS of OH^- signals during the NH_3 transformation over Ru/MgO(100) under different temperatures.

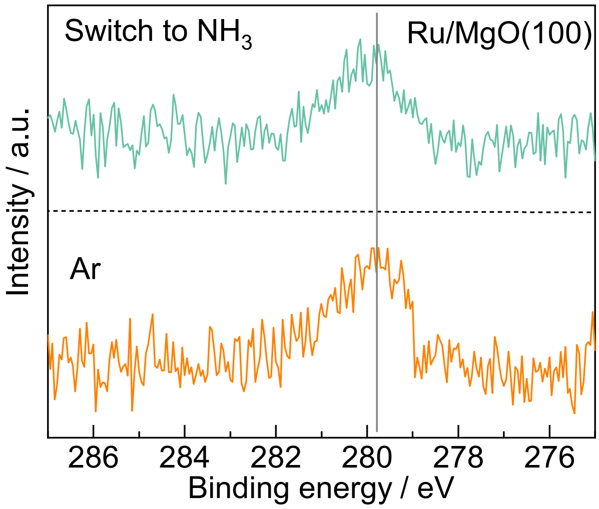

Fig. S13 Ru 3d of AP-XPS spectra at 300 °C over Ru/MgO(100) under alternative sweeping between 0.9 mbar Ar and 0.9 mbar NH_3.

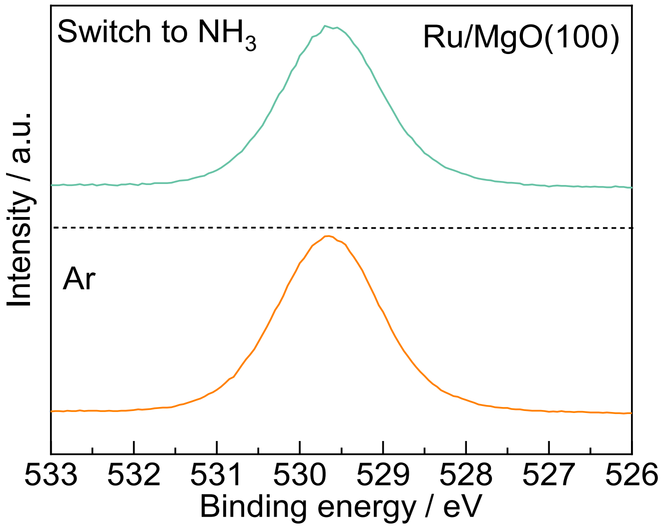

Fig. S14 O 1s of AP-XPS spectra at 300 °C over Ru/MgO(100) under alternative sweeping between 0.9 mbar Ar and 0.9 mbar NH_3.

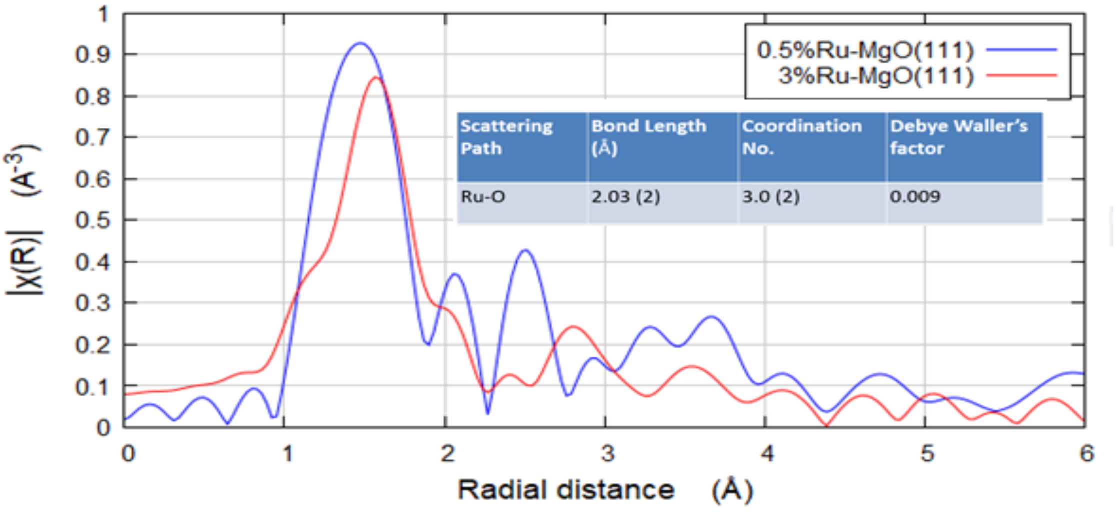

Fig. S15a Fourier Transformed of Ru K-edge EXAFS spectra for Ru/MgO(111) at typical 0.5% and 3% Ru after hydrogen pre-reduction, which show absence of Ru-Ru bond. Parameters obtained from least-square fitting are shown on the right. Fitting parameters for 3% Ru/MgO(111) used: k-range: 3-12, R-range: 1-3, R-factor: 1.9%.

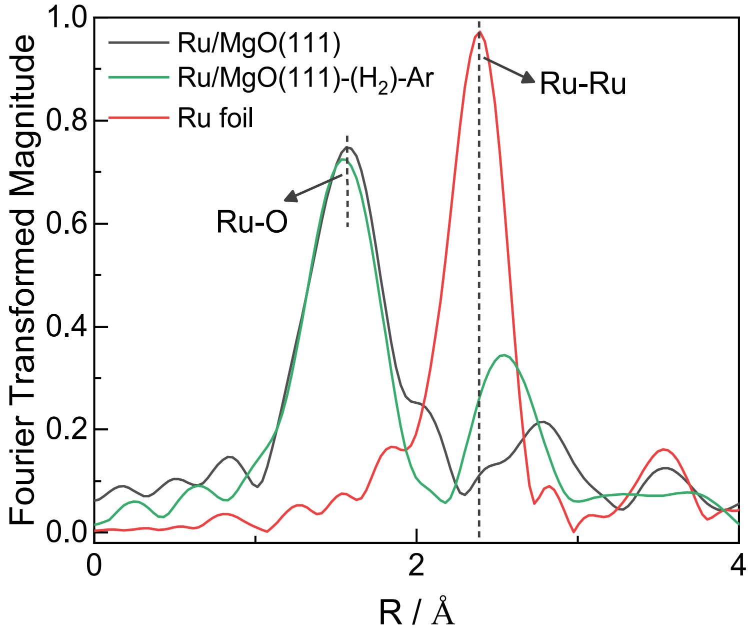

Fig. S15b Fourier transform of k^3-weighted Ru K-edge of EXAFS spectra of Ru/MgO(111) samples after H_2 pre-reduction in Ar. The Ru metal foil is also included here for reference.

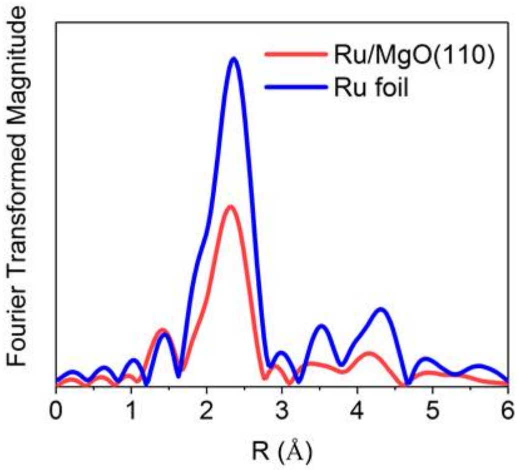

Least square fitting for Ru-MgO(110):

Scattering path	Bond Length (Å)	Coordination No.	Debye-Waller factor
Ru-Ru	2.68 (3)	6.1 (4)	0.004 (1)

Fig. S15c Fourier Transformed of Ru K-edge EXAFS spectra for 3% Ru/MgO(110) or >4% Ru/MgO(111) and Ru(0) foil after hydrogen pre-reduction in Ar, which show presence of extensive Ru-Ru bonds. Parameters obtained from least- square fitting are shown on the right. Fitting parameters for 3% Ru/MgO(110) used: k-range: 3-11.4, R-range: 1.35-3, R-factor: 2.0%.

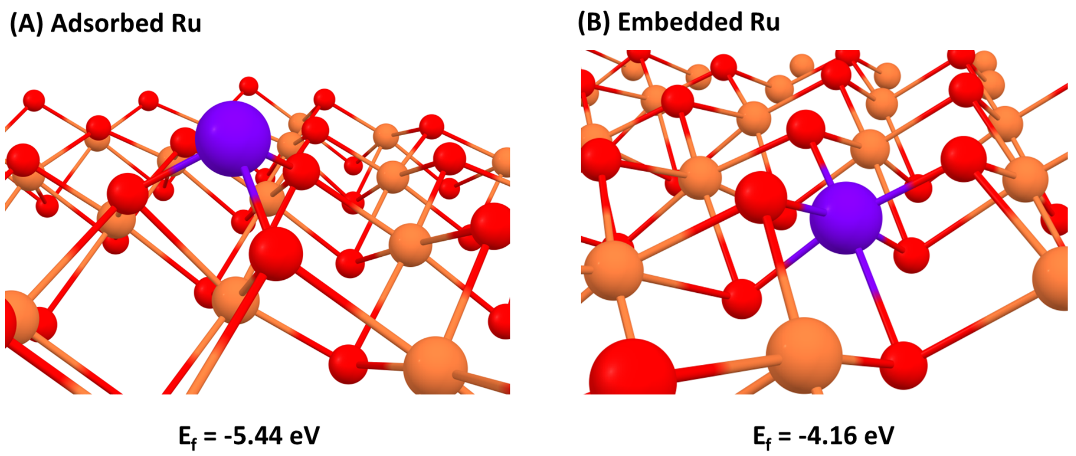

Fig. S16a Optimized structure and formation energies (E_f, in eV) of a single Ru atom (A) absorbed on MgO(111) (O-O-O hollow) site and (B) embedded in place of an Mg atom of MgO(111).

Ru_1 adsorption on nonstoichiometric and stoichiometric MgO(111) surface

System	Ru site	E_{ads}/Ru (eV)	Erel (eV)	Q(Ru) |e|	μ(Ru)	Fig.
stoichiometric	Hollow-top O	-13.35	0.00	1.50	1.52	A
	Hollow-top Mg	-13.17	0.18	1.50	1.47	B
nonstoichiometric	Hollow-top O	-12.95	0.00	1.55	1.56	C
	Hollow-top Mg	-12.63	0.32	1.51	1.49	D

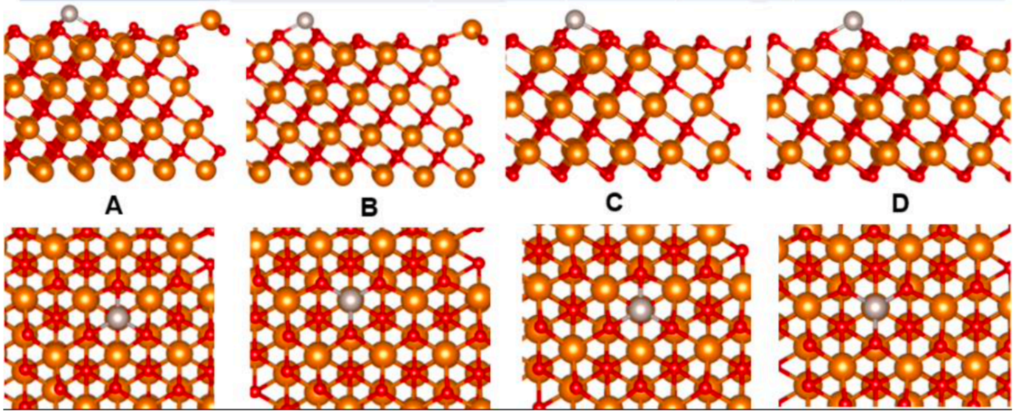

Fig. S16b Comparison between DFT models of Ru atom adsorbing on stoichiometric and non-stoichiometric MgO(111).

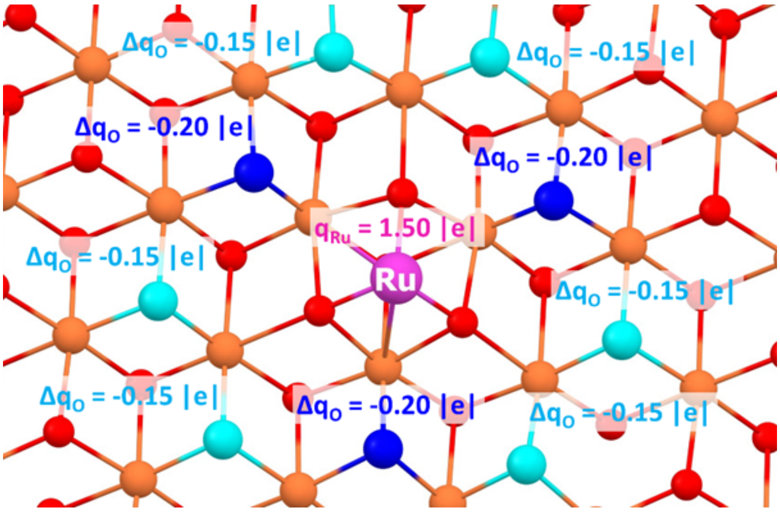

Fig. S16c Bader charge (q, in |e|) of Ru (pink) adsorbed on MgO(111) and the change in Bader charge before and after Ru adsorption (Δq, in |e|) of MgO surface O atoms one bond (blue) and two bonds (cyan) away from Ru.

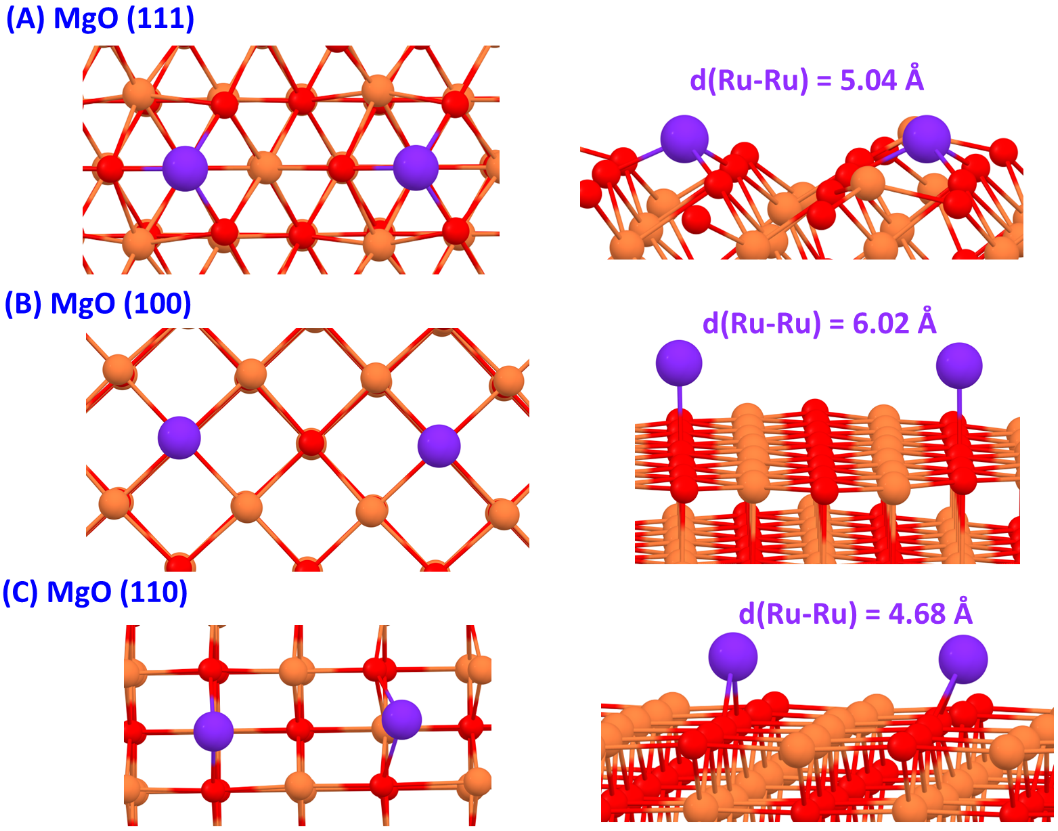

Fig. S17 Optimized structures of atomic Ru pairs adsorbed on the surface of (A) MgO(111), (B) MgO(100) and (C) MgO(110) shown from (left) side view, (right) top view. The shortest distance between two Ru single atoms on each systems are shown. Ru, Mg and O atoms are shown as purple, orange and red spheres.

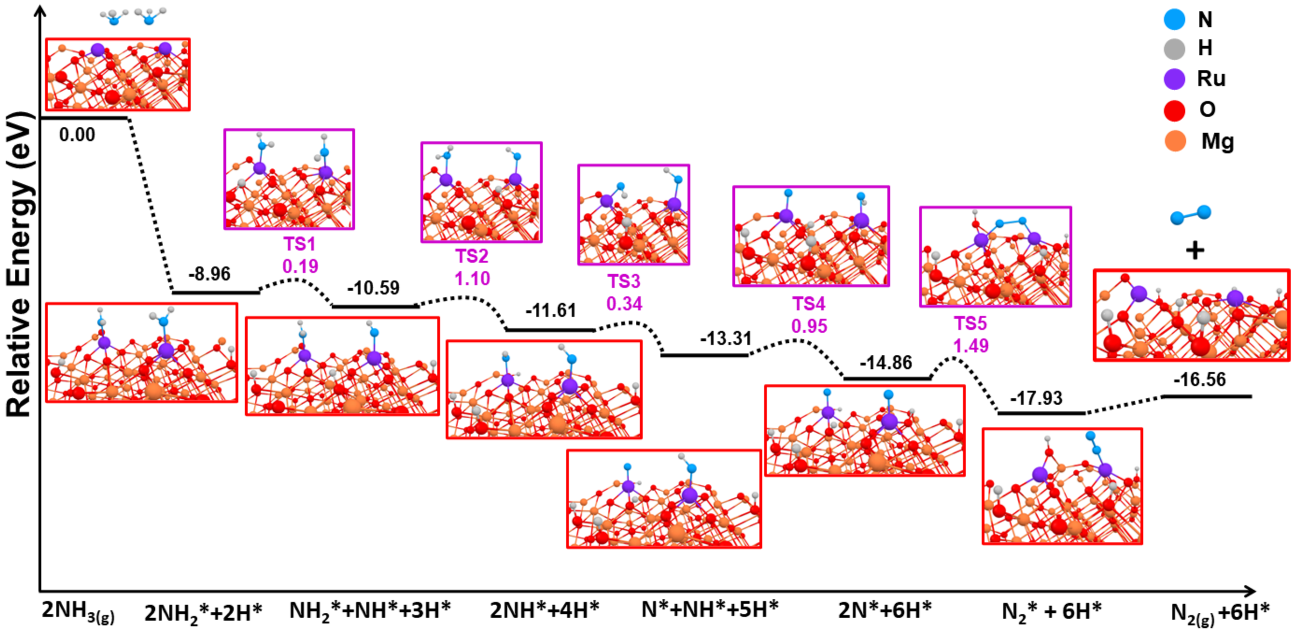

Fig. S18 DFT calculated energy profile of the ammonia decomposition pathway on Ru pair sites supported on MgO(111). The N-N recombination step with a calculated activation energy value of 1.49 eV (TS5) is the rate-determining step.

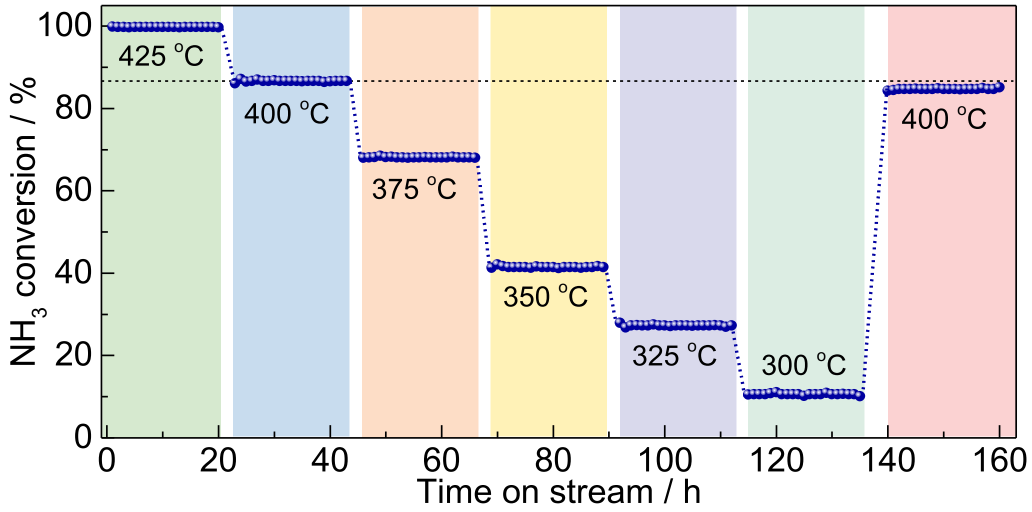

Fig. S19 The catalytic performance of ammonia decomposition and stability of Ru/MgO(111) as a function of step-by-step temperature ramp down and up.

From the figure, it is shown that the Ru/MgO(111) catalyst is stable during the ammonia decomposition. The catalyst was first tested at 425 °C and then the temperature was ramped down step by step. Each step was held on for 20 h on stream. According to the Fig. S19, the Ru/MgO(111) displays a robustness in activity under different temperatures during T-ramp down and no obvious deactivation was observed in our case. It is worth noting that the conversion of ammonia decomposition under different temperatures is similar with the catalytic activity shown in Fig. 2a during T-ramp up. Furthermore, when the temperature was raised up to 400 °C, the ammonia conversion is 84.7%, which is almost the same with the initial activity obtained, further confirming the robustness in activity of the Ru/MgO(111).

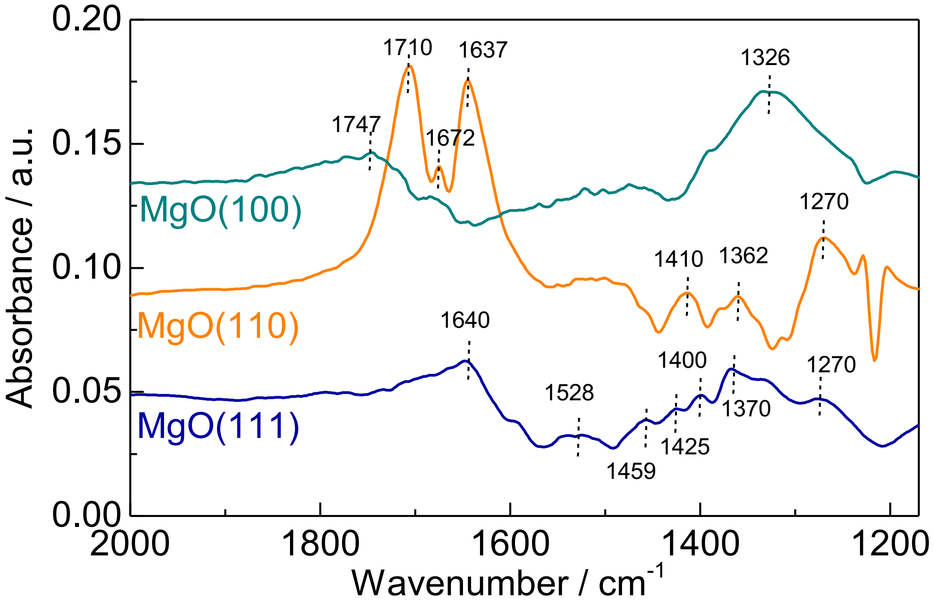

Fig. S20 Recorded DRIFTS of adsorbed species over MgO(111), MgO(110) and MgO(100) after CO_2 adsorption under Ar gas flow.

Table S1 NH_3 conversion over Ru catalysts under atmospheric pressure.

Catalyst	Ru / wt%	T / °C	WHSV / mL g_{cat}^{-1} h^{-1}	Conv. / %	H_2 formation rate / mmol g_{Ru}^{-1} min^{-1}	TOF_{NH3} [e] / s^{-1}	Ref.
Ru/Al_2O_3	10	450	30,000	31.5	115	4.6	1
Ru/SiO_2	10	450	30,000	34.5	114	11.3[f]	1
Ru/MCM-41	5	450	30,000	42.4	284	2.3	2
Ru@ZrO_2	3	450	30,000	ca. 40	447	–	3
Ru/CNTs	5	450	30,000	43.7	292	0.75	4
Ru/MgO	4.8	450	30,000	30.8	215	2.6	5
Ru/TiO_2	4.8	450	30,000	27.2	190	2.1	5
Ru/Al_2O_3	4.8	450	30,000	23.3	163	1.6	5
Ru/AC	4.8	450	30,000	28.7	200	1.7	5
Ru/CNFs	3.2	500	6,500	99.0	206	0.23	6
Ru/Cr_2O_3	5.0	600	30,000	99.9	667.5	0.12	7
Ru/$La_{0.33}Ce_{0.67}$	1.8	450	6,000	100	372	1.8	8
Ru/CeO_2	1.0	350	22,000	ca. 32.0	814	2.13	9
Ru/La_2O_3	4.8	450	18,000	72.8	304.2	1.5	10
Ru-$Mg(NH_2)_2$	5.0	400	60,000	ca. 3~4	24.2	0.09	11
Ru-$Ca(NH_2)_2$	4.6	400	60,000	ca. 7~8	100	0.28	11
Ru-$Ba(NH_2)_2$	4.4	400	60,000	ca. 20	183.4	0.86	11
Ru/C12A7:e^- [a]	2.2	400	15,000	70.0	532	6.9	12
Ru/C12A7:e^- [b]	2.2	450	15,000	ca. 99.9	759	6.9	12
Ru/BHA[c]	2.74	450	60,000	ca. 20.8	507	2.9	13
Ru/MgO	2.8	450	30,000	41.3	493	0.84	14
Ru/MgO[d]	3.5	450	36,000	52.7	606	2.0	15
Ru/MgO-MIL[e]	3.1	450	15,000	ca. 70.0	377	–	16
Ru/c-MgO	4.7	450	30,000	80.6	565	2.6	17
Ru/Com-MgO	3.3	400	30,000	24.8	251.0	0.71	This work
Ru/MgO(100)	3.2	400	30,000	20.1	209.4	0.59	This work
Ru/MgO(110)	3.2	400	30,000	32.2	337.5	0.90	This work
Ru/MgO(111)	0.08	400	30,000	4.5	1902.0	3.43	This work

Ru/MgO(111)	0.94	400	30,000	31.2	1109.9	2.25	This work
Ru/MgO(111)	3.1	400	30,000	68.9	745.2	2.33	This work
Ru/MgO(111)	3.1	450	30,000	100.0	1080.6	4.91	This work
Ru/MgO(111)	3.1	450	60,000	82.2	1777.4	4.91	This work

[a] Reaction conditions: WHSV = 15,000 mL g^{-1} h^{-1}, T = 400 °C; [b]reaction conditions: GHSV = 15,000 mL g^{-1} h^{-1}, T = 450 °C; [c]BHA: Barium hexaluminate, conditions: WHSV = 60,000 mL g^{-1} h^{-1}; [d] reaction conditions: WHSV = 36,000 ml g^{-1} h^{-1}, K/Ru=1/2; [e]conditions: WHSV = 15,000 mL g^{-1} h^{-1}, T = 450 °C; [e] obtained from Ref., estimated based on the published data or calculated by experimental data; [f] the metal dispersion is 1.2%.

Table S2 Least square fitting for Ru/MgO(111) and Ru/MgO(110).

Sample	Scattering path	Bond length / Å	Coordination No.	Debye Waller's factor
Ru/MgO(111)	Ru–O	2.03 (2)	3.0 (2)	0.009
	Ru–Mg	3.17 (3)	1.4 (3)	0.003
Ru/MgO(111)-(H_2)-Ar	Ru–O	2.00 (1)	5.0 (4)	0.006
	Ru–Ru	2.73 (1)	2.6 (9)	0.010
Ru/MgO(110)-(H_2)-Ar	Ru–Ru	2.63 (3)	6.1 (4)	0.004

Table S3 Comparison of Ru/MgO catalysts regarding surface area, catalytic activity: per gram of catalyst and per unit surface area.

	Surface area / m^2/g	Activity /μmol_{NH3} g_{cat}^{-1} s^{-1}	Activity/ surface area /μmol_{NH3} m^{-2} s^{-1}
Ru/MgO(111)	56.2	247.8	4.4
Ru/MgO(110)	38.3	119.8	3.1
Ru/MgO(100)	21.2	74.7	3.5

Table S4 Comparison of kinetic parameters of supported Ru catalysts.

Catalyst	$r = k\, P_{NH3}^{\alpha}\, P_{N2}^{\beta}\, P_{H2}^{\gamma}$				Ea / kJ mol^{-1}
	α(NH_3)	β(N_2)	γ(H_2)	−γ/α	
Ru/MgO(111)	0.66	0	−0.47	0.71	73.0
Ru/MgO(110)	0.49	0	−0.79	1.61	95.5
Ru/MgO(100)	0.2	0	−0.85	4.25	119.2

Table S5 Comparison of activity for ammonia decomposition over atomic dispersed Ru/MgO(111), Ru/MgO(110) and Ru/MgO(100) with an ultra-low loading of 0.01 wt.%.

	Metal loading / wt.%	Activity /μmol$_{NH3}$ g_{Ru}^{-1} s^{-1} [a]
Ru/MgO(111)	ca. 0.01	11160 ± 1000
Ru/MgO(110)	ca. 0.01	1850 ± 300
Ru/MgO(100)	ca. 0.01	1290 ± 400

[a] Based on the metal loading calculated from the ICP-OES results. Reaction conditions: T = 400 °C, WHSV = 30,000 mL g_{cat}^{-1} h^{-1}, 1 bar.

Table S6 Summary of literature experimental attributions of IR bands assigned to carbonates species adsorbed on MgO surface.

Species	Structure	Carbonates			Ref.
		υ_{3high}	υ_{3low}	υ_1	
monodentate	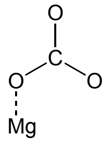	1510-1550	1390-1410	1035-1050	18,19
		1550	1410	1050	
		1520	1730	1060	
		1590, 1510	1415, 1450		
bidentate	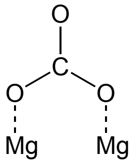 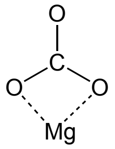		1385, 1335		18,19
		1659,1626	1329, 1273	1024, 947	
		1665-1710	1325-1330	1005-1030	
		1670, 1630	1270		
		1670, 1630	1315, 1280	1000,850,950	
Tridentate	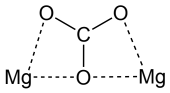	1325-1330, 1745-1750			18,19

Table S7 Summary of the percentage of exposed facets over different MgO samples based on low temperature CO_2 adsorption.

Sample	Peak / cm^{-1}	Facet	Area / %	Facets percentage / %		
				(100)	(110)	(111)
MgO(100)	1747	100	33.9	98.6	1.4	0.0
	1670	100	1.4			
	1326	100	64.7			
MgO(110)	1710	110	43.7	0.0	86.1	13.9
	1637	110	36.8			
	1410	111	5.3			
	1362	110	5.6			
	1270	111	8.6			
MgO(111)	1640	110	23.5	10.1	23.5	66.4

1528	111	4.2
1459	111	4.7
1425	111	6.9
1400	111	3.0
1370	111	30.3
1330	100	10.1
1270	111	17.3

We have cross-checked the total activity of the Ru/MgO (111) nanosheet sample obtained which actually contains (111), (110), and (100) facets in the above percentage by taken the deconvoluted activity of each (100), (110), and (111) facets derived from contour map and total surface area into account. We have found an excellent agreement showing the acceptable quantitative analysis of the facet activity within experimental errors.

Table S8 Rates of ammonia decomposition over the different MgO supported Ru catalysts.

**Sample**	**Ru content / wt.‰** [a]	**CO uptake / μmol g_{cat}^{-1}**	**Dispersion / %**	**Activity /μmol$_{NH3}$ g_{Ru}^{-1} s^{-1}**	**TOF / s^{-1}** [b]
Ru/MgO(111)	0.102	0.68	67.4	11160	1.67
Ru/MgO(110)	0.113	0.58	51.9	1850	0.36
Ru/MgO(100)	0.108	0.49	45.9	1290	0.28

[a]Obtained by ICP-OES analysis; [b] TOF values are based on the CO chemisorption uptake over different samples with <15% conversion; reaction conditions: T=400 °C, WHSV = 30,000 mL g_{cat}^{-1} h^{-1}, 1 bar.

Table S9 Rates of ammonia decomposition over the MgO(111) supported Ru catalysts with different loadings.

Sample	Ru content[a] / wt.‰	CO uptake / μmol g_{cat}^{-1}	Dispersion / %	Activity /μmol NH_3 g_{Ru}^{-1} s^{-1}	TOF[b] / s^{-1}
	0.102	0.68	67.4	11160	1.67
	0.197	1.30	66.5	12091	1.84
	0.309	2.02	66.0	14881	2.28
	0.388	2.51	65.5	18601	2.87
	0.786	4.84	62.3	21159	3.43
	1.05	6.35	61.1	19520	3.23
	1.91	11.26	59.6	15625	2.65
	2.95	17.13	58.7	13481	2.32
	5.1	29.06	57.6	12887	2.26
Ru/MgO(111)	9.4	51.60	55.5	12332	2.25
	20.7	105.65	51.6	11573	2.27
	31.2	150.91	48.9	11281	2.33
	35.5	174.52	49.7	12755	2.59
	41.1	193.10	47.5	13765	2.93
	45.4	210.61	46.9	13228	2.85
	54.3	241.69	45.0	12301	2.76
	61.2	266.96	44.1	10624	2.44
	70.9	306.46	43.7	10204	2.36
	104	398.10	38.7	8229	2.15

[a]Obtained by ICP-OES analysis; [b] TOF values are based on the CO chemisorption over different samples with <15% conversion; reaction conditions: T=400 °C, WHSV = 30,000 mL g_{cat}^{-1} h^{-1}, 1 bar.

Table S9 Quantitative comparison of activity of Ru supported on MgO derived from the contribution of individual facets (via CO_2 adsorption) with the measured value.

Catalyst	Ru / wt%	T / °C	WHSV / mL g_{cat}^{-1} h^{-1}	Conv. / %	Facet percentage / % [a]			H_2 formation rate / mmol g_{Ru}^{-1} min^{-1}	
					(100)	(110)	(111)	By NH_3 Conversion [b]	By individual facet activity [c]
Ru/MgO(100)	3.2	400	30,000	20.1	98.6	1.4	0.0	209.4	215.0
Ru/MgO(110)	3.2	400	30,000	32.2	0.0	86.1	13.9	337.5	388.3
Ru/MgO(111)	3.1	400	30,000	68.9	10.1	23.5	66.4	745.2	745.2

[a] Calculated by the CO_2 adsorption; [b] calculated by ammonia conversion of different samples under 400 °C with a WHSV of 30,000 mL g_{cat}^{-1} h^{-1}; [c] calculated based on the facet percentage of samples according to the CO_2 adsorption; the individual contribution of MgO facets was based on the analysis of 2D contour maps (Fig. 2h).

Reference:

1. Choudhary, T. V. Sivadinarayana, C., & Goodman, D. W. Catalytic ammonia decomposition: CO^x-free hydrogen production for fuel cell applications. *Catal. Letters* **72**, 3–4 (2001).
2. Li, X. K., Ji, W. J., Zhao, J., Wang, S. J. & Au, C. T. Ammonia decomposition over Ru and Ni catalysts supported on fumed SiO_2, MCM-41, and SBA-15. *J. Catal.* **236**, 181–189 (2005).
3. Lorenzut, B. *et al.* Embedded Ru@ZrO_2 Catalysts for H_2 Production by Ammonia Decomposition. *ChemCatChem* **2**, 1096–1106 (2010).
4. Yin, S. F., Xu, B. Q., Ng, C. F. & Au, C. T. Nano Ru/CNTs: A highly active and stable catalyst for the generation of COx-free hydrogen in ammonia decomposition. *Appl. Catal. B Environ.* **48**, 237–241 (2004).
5. Yin, S. F., Xu, B. Q., Zhu, W. X., Ng, C. F., Zhou, X. P., & Au, C. T. Carbon nanotubes-supported Ru catalyst for the generation of CO_x-free hydrogen from ammonia *Catal. Today* **93–95**, 27–38 (2004).
6. Duan, X. Z.; Zhou, J. H.; Qian, G.; Li, P.; Zhou, X. G.; Chen, D. Carbon Nanofiber-Supported Ru Catalysts for Hydrogen Evolution by Ammonia Decomposition. *Chin. J. Catal.* **31 (8)**, 979−986 (2010).

7. Li, L.; Wang, Y. H.; Xu, Z. P.; Zhu, Z. H. Catalytic ammonia decomposition for CO-free hydrogen generation over Ru/Cr_2O_3 catalysts. *Appl. Catal., A* **467**, 246−252 (2013).
8. Le, T. A.; Kim, Y.; Kim, H. W.; Lee, S. U.; Kim, J. R.; Kim, T.W.; Lee, Y.J. & Chae, H. J. Ru-supported lanthania-ceria composite as an efficient catalyst for CO_x-free H_2 production from ammonia decomposition. *Appl Catal B Environ.* **285**, 119831 (2021).
9. Hu, X.C.; Fu, X.P.; Wang, W.W.; Wang, X.; Wu, K.; Si, R.; Ma, C.; Jia, C.J. & Yan, C.H. Ceria-supported ruthenium clusters transforming from isolated single atoms for hydrogen production via decomposition of ammonia. *Appl Catal B* **268**, 118424 (2020).
10. Huang, C.; Yu, Y.; Yang, J.; Yan, Y.; Wang, D.; Hu, F.; Wang, X.; Zhang, R. & Feng, G. Ru/La_2O_3 catalyst for ammonia decomposition to hydrogen. *Appl Surf Sci* **476**, 928–936 (2019).
11. Yu, P.; Guo, J.; Liu, L.; Wang, P.; Chang, F.; Wang, H.; Ju, X. & Chen, P. Effects of Alkaline Earth Metal Amides on Ru in Catalytic Ammonia Decomposition. *The Journal of Physical Chemistry C* **120**, 2822–2828 (2016).
12. Hayashi, F. *et al.* Ammonia decomposition by ruthenium nanoparticles loaded on inorganic electride C12A7:e-. *Chem. Sci.* **4**, 3124–3130 (2013).
13. Wang, Z., Cai, Z. & Wei, Z. Highly Active Ruthenium Catalyst Supported on Barium Hexaaluminate for Ammonia Decomposition to CO_x-Free Hydrogen. *ACS Sustain. Chem. Eng.* **7**, 8226–8235 (2019).
14. Zhang, J., Xu, H., Ge, Q. & Li, W. Highly efficient Ru/MgO catalysts for NH3 decomposition: Synthesis, characterization and promoter effect. *Catal. Commun.* **7**, 148–152 (2006).
15. Ju, X. *et al.* Mesoporous Ru/MgO prepared by a deposition-precipitation method as highly active catalyst for producing CO_x-free hydrogen from ammonia decomposition. *Appl. Catal. B Environ.* **211**, 167–175 (2017).
16. Li, J. *et al.* Sub-nm ruthenium cluster as an efficient and robust catalyst for decomposition and synthesis of ammonia: Break the "size shackles". *Nano Res.* **11**, 4774–4785 (2018).
17. Ju, X. *et al.* Highly Efficient Ru/MgO Catalyst with Surface-Enriched Basic Sites for Production of Hydrogen from Ammonia Decomposition. *ChemCatChem* **11**, 4161–4170 (2019).
18. Y Cornu, D.; Guesmi, H.; Krafft, J.M.; Lauron-Pernot, H. Lewis Acido-Basic Interactions between CO_2 and MgO Surface: DFT and DRIFT Approaches. *J. Phys. Chem. C* **116**, 6645−6654 (2012).
19. Y Mutch, G.A.; Shulda, S.; McCue, A.J.; Menart, M.J.; Ciobanu, C.V.; Ngo, C.; Anderson, J.A.; Richards, R.M.; Vega-Maza, D.; *J. Am. Chem. Soc.* **140**, 4736−4742 (2018).
